# Size at birth and cognitive ability in late life: A systematic review

**DOI:** 10.1002/gps.5138

**Published:** 2019-06-13

**Authors:** Murali Krishna, Steven Jones, Michelle Maden, Bharath DU, Ramya MC, Kalyanaraman Kumaran, Samuel Christraprasad Karat, Caroline H.D. Fall

**Affiliations:** ^1^ Department of Research Foundation for Research and Advocacy in Mental Health (FRAMe) Mysore India; ^2^ Epidemiology Research Unit CSI Holdsworth Memorial Hospital Mysore India; ^3^ Medical Institute, Riverside Campus University of Chester Chester UK; ^4^ Post Graduate Medical Institute, Faculty of Health and Social Care Edge Hill University Ormskirk UK; ^5^ Jerudong Park Medical Centre Jerudong Brunei; ^6^ MRC Lifecourse Epidemiology Unit University of Southampton Southampton UK

**Keywords:** birth weight, cognition, DOHaD, systematic review

## Abstract

**Introduction:**

Recent evidence suggests that growth restriction in utero may lead to neurocognitive disorders in late life, either through impaired brain development or adverse metabolic programming.

**Methods:**

Systematic review of literature investigating the relationship between size at birth and cognitive abilities in late life. The search, data extraction, and rating for the quality of reporting were conducted independently by two researchers.

**Results:**

Of 533 selected studies, 11 were included in this systematic review and 10 of these were from high‐income setting. Of these 11 studies, eight indicated that lower birth weight is a risk factor for lower cognitive function in late life, at least in high‐income countries. The reported effect sizes were small and it was not possible to conduct meta‐analyses because of clinical heterogeneity

**Discussion:**

A modest association of lower birth weight with lower cognitive abilities in late life is consistent with persisting effects of the prenatal environment on brain function. As with all observational studies, confounding is an alternative explanation. Further studies are required to elucidate the mechanisms.

**Key points**

Recent evidence suggests that growth restriction in utero may lead to neurocognitive disorders in later life, either through impaired brain development or adverse metabolic programming. A modest association of lower birth weight with lower cognitive abilities in later life, observed in this systematic review, is consistent with persisting effects of the prenatal environment on brain function in later life.

**Abbreviations**
AH4: Alice Heim Test Version 4
BCS: British cohort study
CHD: coronary heart disease
CINAHL: Cumulative Index to Nursing and Allied Health Literature
COWAT: Controlled Word Association Test
DOHaD: Developmental Origins of Health and Disease
HIC: higher income country
LMIC: low‐ and middle‐income country
MCS: Millennium Cohort Study
MMSE: Mini Mental State Examination
NART: National Adult Reading Test
NCDS: National Child Development Study
PRISMA: Preferred Reporting Items for Systematic Reveiws and Meta‐Analyses
SEP: socioeconomic position
STROBE: Reporting of OBservational Studies in Epidemiology

## 1 BACKGROUND

Neurocognitive disorders are a major cause of disability and mortality in late life and are associated with high costs for health systems and society.^1^, ^2^ For late‐life neurocognitive disorders, as for other late‐life chronic diseases, there is renewed interest in the relevance of DOHaD hypothesis with two plausible pathways to cognitive ageing: (a) by a direct effect of reduced intrauterine nutrition (reflected in birth size) on fetal brain development leading to reduced cognitive reserve and decreased cognitive ability or (b) programming of metabolism in very early life by under‐nutrition, leading to increased risk mediated through cardiometabolic disorders.^3^

Quality of nutrition during intrauterine development, reflected crudely in size at birth, is an important determinant of lifelong function, health, and disease risk.^4^ Birth weight and head circumference at birth are indicators of intrauterine growth and brain development, respectively.^5^ Larger birth weight, the most widely researched birth size measure, is associated with better cognitive function and higher intelligence from infancy through the third decade of life in several populations and countries independent of social background.^6^, ^7^, ^8^ This association of birth weight with cognition occurs across the whole spectrum of birth weight rather than being confined to an extreme group. However, the strength of this association is known to diminish as individuals reach middle age, and associations with growth in early life may not persist beyond midlife.^8^

In a systematic review conducted in 2015, Grove and colleagues examined the relationship between birth weight and general cognitive ability in non‐clinical adult populations.^8^ This included 1 122 858 participants aged between 18 to 78.4 years from 19 studies. Of these, only eight could be included in a random‐effects meta‐analysis and three were in those aged 60 yrs and above. There was a modest association of birth weight with cognitive ability; with each kilogramme increase in birth weight, there was a 0.13 SD increase in general intelligence (95% CI, 0.07,‐0.19) in those aged less than 60 yrs, independent of gestational age and parental social class at birth. However, the effect size was much lower and not statistically significant in those aged 60 years and above (0.07 SD; 95% CI, −0.02 to 0.16). In addition to the small number of studies, the authors did not consider other birth size parameters (like head circumference, length at birth, and ponderal index), which are known to be associated with cognitive ability in this age group.^9^, ^10^, ^11^ While birth weight was not a reliable predictor of cognitive ability or decline beyond midlife in this review, it would be premature to conclude that prenatal environment is not associated with cognitive ability in late life.

## 2 AIMS

The aim of this systematic review was to locate, appraise, and synthesise studies investigating the relationship between size at birth and cognitive ability in late life.

## 3 MATERIALS AND METHODS

It was conducted according to the Cochrane guidelines for systematic reviews of observational studies and adheres to the Preferred Reporting Items for Systematic Reviews and Meta‐Analyses (PRISMA) guidance.^12^

### 3.1 Inclusion and exclusion criteria

Cross‐sectional or longitudinal studies examining the relationship between *any* birth size parameter (birth weight, birth length, head circumference, and ponderal index) and performance on *any* cognitive function test in adults aged 50 years and above were eligible for inclusion. Studies were excluded if they examined the association of birth size with mental disorders (eg, depression) or physical health (eg, frailty) without reporting measurements of cognitive performance or were purely qualitative in nature.

### 3.2 Identification and selection of studies

**Figure 1:**
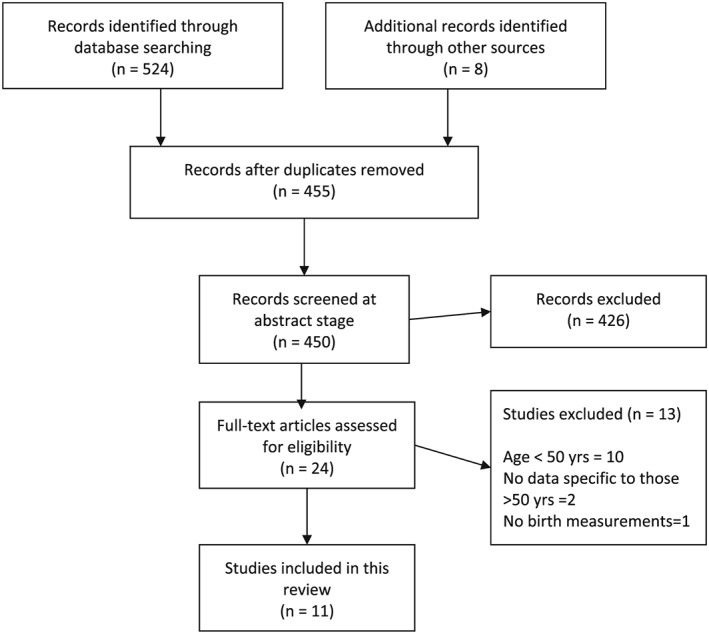
Flow diagram illustrating the process of selection of eligible studies for this systematic review

Searches were undertaken by three independent researchers (M.K., B.D.U., and M.M.) in the following databases: MEDLINE, Embase, PsychINFO, and CINAHL. Databases were searched from their inception to February 2019. Two reviewers (M.K. and S.J.) independently screened all the potential studies against the inclusion criteria. Disagreements were resolved by discussion. The population search terms (both MeSH terms and text words) for *exposure* included “birth weight, birth size, birth length, ponderal index, growth in utero, fetal growth, fetal development, fetal growth retardation, intrauterine growth, prenatal nutrition, and fetal origins hypothesis,” and for outcome included “cognition, memory, attention, recall, intelligence, brain function, and dementia.” Where available, limits appropriate to participants (human studies), age (above 50 years), and study design (cohort studies, observational studies, and longitudinal studies) were applied. No date or language restrictions were applied. The search strategy from one of the engines (MEDLINE) is provided as an appendix (Appendix A). Experts in the field were contacted for any ongoing and unpublished studies. Authors were contacted for additional information when indicated. Reference lists of included studies were scanned for additional relevant publications. Citation searches were also conducted on key papers. The *International Journal of Geriatric Psychiatry*, *Journal of Alzheimer's Disease and Dementia*, and *Journal of Developmental Origins of Health and Disease* were manually searched from March 2015 to February 2019 (Figure 1).

### 3.3 Data extraction and analyses

A data extraction form was created and piloted. Data were extracted on all measurements of size at birth, scores on cognitive function tests (both for individual domains and composite scores), and any other relevant key data. The quality of eligible studies was evaluated using the Strengthening the Reporting of OBservational Studies in Epidemiology (STROBE) checklist.^13^ Two independent researchers (M.K. and S.J.) undertook data extraction and quality assessment. Disagreements were resolved by consensus.

If it was feasible to conduct a meta‐analysis, it was planned to provide an estimate of combined effect size. If sufficient numbers of eligible studies were retrieved, it was planned to evaluate publication bias by a funnel plot analysis.

## 4 RESULTS

### 4.1 Selection of studies

Selection process for this systematic review was conducted in accordance with the PRISMA guidelines.^12^ Figure 1 outlines the results of the search process. Of the 533 selected studies, 11 met the eligibility criteria for this review.^9^, ^10^, ^11^, ^14^, ^15^, ^16^, ^17^, ^18^, ^19^, ^20^, ^21^

### 4.2 Key characteristics

#### 4.2.1 Setting and design

The studies were published between 1996 and 2014 and included community‐dwelling men and women who volunteered to participate. Two studies had a cross‐sectional design^11^, ^14^ while others were longitudinal follow‐ups of established cohorts.^9^, ^10^, ^15^, ^16^, ^17^, ^18^, ^19^, ^20^, ^21^ Of the 11 studies, nine were cohort studies in which participants were matched to their birth records. The other two were community‐based cohorts from the United States. Set up for examining cardiovascular disorders and birth weight was self‐reported by the participants (Table 1).

**Table 1:** Key characteristics of the studies included in this systematic review

First author, Year, and Country	Population and Setting	Study Design	Sample Size, Gender, and Age	Exclusion Criteria	Early Life Exposures	Cognitive Outcomes
Martyn 1996 UK	Men and women born in Hertfordshire, Sheffield, or Preston between 1920 and 1943	Longitudinal follow‐up of a birth cohort.	N = 1576 (% F unclear) Mean 61 (2.1) yr	Those born before 38 weeks of gestation.	Birth weight, length, head circumference, gestational age, maternal age, parity, and paternal occupation	Alice Heim intelligence test and Mill Hill Vocabulary test
Raikkonen 2013 Finland	Men born Helsinki between 1934 and 1944 and performed compulsory military service.	Longitudinal follow‐up of the Helsinki birth cohort.	N = 931 (0% F) Mean 68 (2.5) yr	Those not living in Helsinki.	Birth weight, length, head circumference, gestational age, maternal age, parity, and height	Finnish Defense Forces basic Intellectual Ability Test
Shenkin 2007 UK	Men and women born in one hospital in Edinburgh UK between 1921 and 1926	Longitudinal follow‐up of a birth cohort.	N = 130 (71% F) Mean 78.4 (1.4) yr 75‐81 yr	Dementia and deafness.	Birth weight, length, head circumference, gestational age, maternal age, parity, and height	Controlled Word Association Test, Murray House Test, Raven's Matrices Test, and National Adult Reading Test.
Gale 2003 UK	Men and women born in Jessop Hospital for Women in Sheffield.	Longitudinal follow‐up of a stratified sample of a birth cohort.	N = 215 (46% F) Mean 70 (2.0) yr 66‐75 yr	Dementia or deafness	Birth weight, length, head circumference, gestational age, and parental occupation	Alice Heim Intelligence Test and Weschler logical memory test
Costa et al 2011 USA	Men and women from Minneapolis and Washington.	Longitudinal follow‐up of a community cohort.	N = 6785 (56% F) Mean 59.2 (5.6 yr) 54‐73 yr	CHD, CVA mental disorders, prematurity, and non white.	Birth weight by recall and non‐hospital records.	Delayed Word Recall test, Digit Symbol Test, and Word Fluency Test
Skogen 2013 Norway	Men and women from Bergen born between 1925 and 1927	Longitudinal follow‐up of a birth cohort.	N = 346 (55% F) 72‐74 yr	Non‐reported	Birth weight, length, head circumference, maternal age and parity, parental occupation	Kendrik Object Learning test, Trail making test, Digit Symbol Test, Block Design, and Controlled Word Association Test.
Hyvarinen 2009 Finland	Men and women living in Helsinki and matched to birth records.	Longitudinal follow‐up of a randomly selected subsample in a birth cohort.	N = 1243 (53% F) 60‐66 yr	Major physical disabilities and poor vision.	Birth weight	Beck's Depression Inventory Battery of cognitive tests (for reaction time, attention, working memory, and associate learning)
Zhang 2009 China	Men and women born in Beijing between 1921 and 1954, and matched to birth records.	Retrospective birth cohort, cross‐sectional design	N = 2062 (48% F) 50‐82 yr	None reported	Birth weight, length, head circumference, maternal age and parity, gestational age, and parental occupation at birth	Fluid object memory test Verbal fluency Weschler intelligence test
de Rooij 2010 Netherlands	Men and women born between 1944 and 1945 (from the Dutch Famine Birth Cohort)	Longitudinal follow‐up of a birth cohort.	N = 737 (53% F) Age 56‐59 yr	Mental disorders	Birth weight, head circumference, gestational age, placental area, and occupation of head of the household	Alice Heim test, Stroop test, Paragraph Encoding and Recall Mirror drawing test
Muller 2014 Iceland	Men and women from Reykjavik born between 1907 and 1935	Longitudinal follow‐up of a randomly selected subjects from a birth cohort.	N = 1254 (57% F) Mean 76 (5) yr 69‐81 yr	Dementia and prematurity	Birth weight and length	California Verbal Learning Test Figure Comparison Test. Digit symbol and Stroop Test, and Spatial Working Memory test.
Ericson 2010 US	Women living in Rancho Bernado	Cross‐sectional	N = 292 55‐89 yr Median 71 yr	Not reported	Birth weight (self reported)	Blessed Dementia Scale, Trail Making Test, Verbal Fluency, and Heaton Visual Memory Test

#### 4.2.2 Demographics

The sample size ranged from 130 to 6875 and participants were aged between 50 to 89 yrs. While Raikkonen et al included men only, Erickson et al included women only.

#### 4.2.3 Factors at birth

Birth weight was a universally available measurement of birth size across all the studies. In two studies^14^, ^15^ both from the USA, birth weight was obtained by recall and non‐hospital records (such as family diaries and birth certificates), and did not provide any other information related to birth. All other studies were based on the birth weight obtained from obstetric records. As a measurement of birth size, only birth weight was available from obstetric records in Hyvarinen et al, while Muller et al had an additional measurement of length at birth. In addition to birth weight, length at birth, head circumference, and gestational age were available from the maternity records in other studies.^9^, ^10^, ^11^, ^16^, ^17^, ^18^

Parental occupation as an indicator of socioeconomic position at birth was available from obstetric records in some studies,^9^, ^11^, ^16^, ^19^ while occupation of the head of the household was available from maternity records from de Rooij 2010 et al. Information about parental education at birth, an important determinant of growth and development of the offspring, was not available in any of the studies.

#### 4.2.4 Cognitive outcomes

All studies examined memory and attention, while most studies (n = 9) had a measure of verbal fluency as cognitive outcomes (Tables 1 and 2). Additional cognitive domains were examined in most of the studies. They include: logical, verbal, and numerical reasoning in Martyn et al; processing speed and executive function in Muller et al; general intelligence and selective attention in de Rooij et al; processing speed, selective attention, visuospatial performance, and motor skills in Skogen et al; verbal, arithmetic and visual spatial reasoning in Raikkoken et al; visuospatial tracking and attention in Erickson et al; verbal and non‐verbal reasoning and executive function in Shenkin et al 2009; intelligence in Zhang et al; reaction time and attention in Hyvarinen et al; and intelligence in Gale et al. None of the studies had cognitive impairment and dementia as outcomes, while, Hyvarinen et al had a measure depressive symptoms.

**Table 2:** Summary of cognitive function tests, effect sizes, and risk of bias in studies included in the systematic review

Study (yr) Birth parameter (units)		Unadjusted correlation			Adjusted correlation			Confounders	Risk of Bias
	Cognitive test	Coefficient	SE	p	Coefficient	SE	P		
Zhang et al (2009) Ponderal Index (kg/m^3^)	Immediate Recall			NR	NR	NR	.50	Gestational age, parity, and paternal occupation at birth; drinking milk during childhood; age, sex, cardiometabolic risk factors, socioeconomic position, and occupation in adult life.	Low
	Delayed Recall			NR	NR	NR	.77		
	Cumulative score	OR = 1.5^a^	NR	0.02	OR = 1.26	NR	.30		
de Rooij et al.(2010)^b^ Birth weight (gms)	Alice Heim (reaction time)	*ρ* = 0.03		ns	NR	NR	NR	No adjustments were made. Spearman correlation coefficients were reported for birth weight and cognitive outcomes.	Medium
	Alice Heim score	*ρ* = 0.06		ns	NR	NR	NR		
	Stroop test	*ρ* = −0.01		ns	NR	NR	NR		
	Stroop score	*ρ* = 0.03		ns	NR	NR	NR		
	Memory Immediate recall	*ρ* = 0.01		ns	NR	NR	NR		
	retrieval	*ρ* = −0.02		ns	NR	NR	NR		
	Mirror errors	*ρ* = −0.07		ns	NR	NR	NR		
	Mirror rounds	*ρ* = 0.06		ns	NR	NR	NR		
	Mirror errors per rounds	*ρ* = −0.08		ns	NR	NR	NR		
Costa et al (2011) Birth weight (gms)	Word fluency			NR	*β* = .752	0.3	.004	Age, sex, education, race, social class, education, smoking, alcohol, body mass index (BMI), and self‐reported cardiometabolic risk factors (diabetes, hypertension, LDL, and HDL cholesterol), and history of stroke.	High
	Delayed word recall			NR	*β* = .028	0.03	ns		
	Digit symbol			NR	*β* = −.067	0.25	ns		
Martyn et al. (1993) Head circumference (inch)	Alice Heim Test			NR	NR	NR	.008	Social class at birth, age, sex and for individual datasets.	Medium
	Decline			NR	NR	NR	.85		
Hyvarinen et al (2009) Birth weight (kgs)	Divided attention			NR	*β* = −3.8	1.38	.005	Gestational age, sex, age, and education (history of heart disease, depression, and self‐reported health status also considered but not included in adjusted model)	Low
	Association learning			NR	*β* = −1.5	0.71	.04		
	Association learning			NR	NR	NR	ns		
	Simple reaction time			NR	NR	NR	ns		
	Choice reaction time			NR	NR	NR	ns		
	Working memory hit rate			NR	NR	NR	ns		
	Working memory reaction time			NR	NR	NR	ns		
Raikkonen et al. (2013) Birth weight (SD)	IQ (Finnish Defense Forces)	*β* = 1.04	0.51	0.04^a^	*β* = 1.31	0.64	.04^a^	Gestational age and parity at birth; breastfeeding in childhood; education, social class, height, and history of heart disease and stroke	Medium
	Decline	*r* = 0.07	0.04	0.04	*r* = 0.08	0.04	.06		
Erickson et al (2010) Birth Weight (lbs)	Buschke total			NR	*β* = ‐−.08		.77	Age and education	Medium
	Buschke LTM			NR	*β* = −.08		.83		
	Buschke STM			NR	*β* = .00		.97		
	Heaton visual copying			NR	*β* = .05		.63		
	Heaton visual LTM			NR	*β* = −.00		.99		
	Heaton visual STM			NR	*β* = .07		.22		
	MMSE total			NR	*β* = .03		.57		
	Serial 7's			NR	*β* = .08		.04^a^		
	world backward			NR	*β* = −.00		.89		
	Trails B			NR	*β* = 2.23		.18		
	Category fluency			NR	*β* = .08		.59		
	Blessed			NR	*β* = .05		.16		
Skogen et al (2013) Birth weight (kgs)	Mini Mental State Examination	*β* = −.03	0.09	ns	*β* = .05	0.09	ns	Age and sex	Medium
	Digit symbol	*β* = −.12	0.44	ns	*β* = −.03	0.45	ns		
	Kendrick	*β* = −.24	0.79	ns	*β* = −.14	0.78	ns		
	Object learning COWAT	*β* = .85	0.55	ns	*β* = .91	0.55	ns		
	Trail making A	*β* = 2.44	2.94	ns	*β* = 2.01	2.97	ns		
	Block Design	*β* = −.23	0.21	ns	*β* = −.26	0.21	ns		
	Composite score	*β* = .01	0.1	ns	*β* = .02	0.1	ns		
Muller et al (2014)^c^ Ponderal Index (Kg/m^3^)	Memory			NR			NR	Age and sex	Medium
	Processing speed			NR	*β* = −.012	NR	.008		
	Executive function			NR	*β* = −.08	NR	.04		
Gale et al (2003) Head circumference at birth (cms)	Alice Heim Intelligence score			NR	NR	NR	.38	Social class at birth, age, sex, education, history of cerebrovascular disease and Nottingham Health Profile emotion subscale	Medium
	Weschler Immediate Recall			NR	NR	NR	.75		
	Weschler Delayed Recall			NR	NR	NR	.74		
	Decline on Alice Heim Intelligence score			NR	NR	NR	.94		
Shenkin et al (2009) Birth weight (gms)	Raven's Progressive Matrices	*r* = 0.15	ns	ns	*r* = 0.08	ns	ns	Gestational age and parity at birth Age, sex, and social class	Low
	Moray House test	*r* = 0.15	ns	ns	*r* = 0.10	ns	ns		
	Test no 12	*r* = 0.08	ns	ns	*r* = 0.03	ns	ns		
	Verbal Fluency	*r* = 0.09	ns	ns	*r* = 0.04	ns	ns		
	g (General Intelligence)	*r* = 0.15	ns	ns	*r* = 0.12	0.27	.27		
	National Adult Reading Test	*r* = 0.10	ns	ns	*r* = 0.15	0.19	.19		
	g corrected for National Adult Reading Test	*r* = 0.10	ns	ns	*r* = 0.15 *r* = 0.05	0.19 0.63	.19.63		

Abbreviations: *β* = effect size from regression analyses; *ρ*, correlation coefficient; NR, not reported; ns = not significant but values not provided; OR, odds ratio; r, rho.

a Odds ratio for lower cognition defined as cumulative score lower than 10 percentile.

b values only for those exposed to famine in utero.

c values for those with low education only.

#### 4.2.5 Confounding factors

The association of birth size with cognitive outcomes was adjusted for a range of confounding factors in most of the studies (Table 2). They include: gestational age, maternal age and parity, indicators of socioeconomic position at birth, attained educational level, social class of participants, and cardiometabolic risk factors. However, these studies do not provide information as to why these factors were thought to be confounding and/or were important as covariates.

#### 4.2.6 Estimates of effect sizes and analyses

The strength of association of birth size parameters with cognitive outcomes was examined and reported differently across studies (Table 2). In addition, many of the eligible studies were relatively small; from diverse population groups, both exposures and outcome measures for cognitive function were multiple and heterogeneous (Table 2). Therefore, it was not possible to conduct a metanalysis or evaluate for publication bias.

### 4.3 Quality of reporting and risk of bias

The quality of reporting of the studies as assessed by the STROBE check list was good to excellent. At least 18 of the 22 items (range 18 to 22) from this checklist were reported (Appendix B). None of the authors reported how the study size was derived. While some (n = 4) did not report the efforts made to address potential sources of bias, some (n = 3) did not discuss the generalisability (external validity) of the study results. Degree of overall bias as estimated from the STROBE check list for individual studies is provided in Table 2. The risk of bias was high in Costa et al primarily because of relatively huge losses to follow‐up and mutliple testing, and low in Zhang et al, Hyvarinen et al, and Shenkin et al. The risk of bias was medium in other studies (Table 2).

### 4.4 Important studies that were excluded

1. Aroujo et al (2014) conducted cognitive assessments of 12 997 men and women aged 35 to 64 yrs from the Brazil Longitudinal Study of Ageing, nearly half of them were aged above 50 years.^22^ Birth weight (self reported) was directly associated with cognitive abilities in this study. However, the authors were unable to provide data specific to those aged 50 yrs and above.
2. Melrose et al (2013) examined the relationship between early life environment and cognitive abilities in 333 men and women from the UC Davis Diversity Ageing Cohort in the United States.^23^ This study was excluded as authors did not specifically report the association of size at birth with cognitive abilities.
3. Richards et al (2001) reported the relationship between birth weight and cognitive function in the British 1946 birth cohort.^24^ Participants were 43 yrs of age when examined and therefore excluded from this review.
4. Dawes et al (2015) examined the effect of prenatal and childhood development on hearing, vision, and cognition in the UK Biobank Cohort.^25^ Participants were aged between 40 to 66 yrs, and birth weight was self‐reported. Authors were contacted and they were unable to provide data specific to those aged 50 yrs and above.

## 5 DISCUSSION

### 5.1 Key findings

Studying early determinants and predictors of cognitive ageing has been repeatedly identified as a research priority.^26^, ^27^ The studies evaluated in this systematic review have contributed significantly to this research and suggest that cognitive function in late life is influenced by nutrition and environment in early life. A majority of the studies (7 of the 11) included in this review indicate that intrauterine growth restriction, crudely reflected in size at birth, is directly associated with lower cognitive ability in late life, at least in high‐income country settings. The overall effect sizes were small and there was insufficient adjustment for important confounders in several studies. It was not possible to compare and appraise the effect sizes of studies with each other or conduct a meta‐analysis to derive a pooled effect size. This was because the associations of different birth size parameters with multiple cognitive outcomes for different domains have been reported and the strength of associations has been reported differently.

The association of birth size with late life cognition was independent of parental socioeconomic position at birth in most studies^15^, ^16^, ^17^, ^18^, ^19^ and was confounded by socioeconomic position at birth in one study.^11^ Parental socioeconomic position at birth was not associated with cognitive function in late life in Shenkin et al, while this association was not examined in the remaining studies.^9^, ^14^, ^20^, ^21^

Across all the studies, adjusting for education attenuated the strength of association of birth size with late life cognition. When reported separately, higher level of attained education was directly associated with higher scores for certain cognitive abilities. A possible mediating or confounding effect of cardiometabolic disorders on the relationship between size at birth and late life cognition was evaluated in three studies included in this review: the direct association of size at birth with late life cognition was independent of stroke and coronary heart disease (CHD) in Raikkonen et al, diabetes and hypertension in Costa et al, and diabetes and CHD in Hyvarinen et al. However, these studies did not examine if smaller size at birth was associated with an increased the risk of cardiometabolic disorders (as potential confounders).

The presence of a relationship between birth parameters and late life cognitive ability does not necessarily imply a direct causal relationship; birth parameters may merely reflect underlying influences. Residual confounding is a major possible reason for any false positive associations. The mechanism of any influence of birth parameters on cognitive ageing has not yet been established, and this may be a direct or an indirect influence through cognitive reserve and cardiometabolic pathways respectively.^3^, ^28^ The studies in this review were not designed to examine the DOHaD pathways of cognitive ageing. Such a study would have examined the association of size at birth with cognitive reserve and/or cardiometabolic risk factors in adult life and, in turn, association of these with cognitive function in late life.

Cognitive decline is thought to begin as early as 40 years of age.^29^ Most studies in this review conducted baseline cognitive assessments when participants were well above the age of 50, by which cognitive decline may already be evident and observed associations (or a lack of) in these studies may be due to a horse racing effect.^30^

While examining cognitive function in the studies included in this review, cognitive decline may have been measured, and mostly the papers were uninformative about this. However, cognitive decline was specifically measured in four studies in this review. Of those that examined the relationship between birth size and cognitive decline, no association was reported in three studies,^9^, ^10^, ^16^ while in one study,^17^ men with larger size at birth had slower rate of cognitive decline in late life.

Findings from this review also suggest that the relationship of growth and development in early life is more likely with cognitive abilities that are associated with the fronto‐temporal lobes of the brain such as verbal fluency, attention, trail making, calculation, executive functioning, and working memory. Of these, verbal fluency is regarded as an index of crystallised intelligence, while others are generally considered as components of fluid intelligence. In fact, the verbal fluency test is particularly sensitive to linguistic impairment and early mental decline in older persons; it is also a sensitive indicator of damage to the left lateral lobe.^31^, ^32^

### 5.2 Strengths and limitations of included studies

The studies that reported a positive association of size at birth with late life cognitive ability generally included relatively well‐educated, predominantly white, and middle‐class men and women from higher income settings, which limits the generalisability of the findings beyond these settings. Moreover, the estimates of the effect sizes reported are at best modest. It is possible that the results are specific to the cohorts under study (cohort bias). These individuals have seen substantial changes in both prenatal and later health care.

None of the studies included in the review have information of the entire eligible population to assess the degree of potential bias. The studies used volunteers, who generally have higher cognitive ability and social class than non‐volunteers.^33^ As all analyses were performed within the study sample, unless the correlation between birth size and cognitive ability differs between the volunteers and non volunteers, it is unlikely that significant bias would have been introduced. Birth weight in the United Kingdom, the United States, and Scandinavian countries, where these studies were conducted, is among the highest in the world, and they also have higher rates of literacy in comparison to LMIC settings.^26^ It is reasonable to postulate that the effect size would be different when studying the relationship between birth size and late life cognition in LMIC populations with proportionately lower birth size and literacy levels.

Attrition bias may have also affected results. Most studies do not provide details about losses to follow‐up. When reported, those who were lost to follow‐up had lower attained education level when compared with those who were re‐examined, this bias may have influenced findings towards non‐significant results.

As is common with longitudinal studies of older adults, participants who were lost to follow‐up in Shenkin et al and Raikkoken et al had lower mean cognitive scores at baseline than those who took part in the repeat testing. Such attrition may induce bias in the estimates of cognitive change. These two studies examined decline based on cognitive data only at two points in time. Random variation or regression to the mean may account for some of the observed changes in cognitive test scores and the results need to be treated with caution.

Participant exclusion is also known to introduce bias. Although most studies in this review excluded a minimal number of participants (Table 1), one study^15^ excluded 36.6% (n = 3921) of participants examined at the initial visit and such an extensive exclusion may limit generalisability to the wider population. In two of the studies,^14^, ^15^ both from the United States, birth weight was obtained by recall and non‐hospital records like birth certificates and family diaries. A problem with this is a possible greater inaccuracy of birth weight recall in those with lower cognitive functioning. In fact, in Costa et al, poorer performance in cognitive tests was observed in those who recalled their birth weight when compared with those with available birth records. This was not examined in Erickson et al, as only a small proportion of those recruited in this study had documented birth weight.

When birth size data were extracted from routinely recorded measurements from historic maternity records, it is possible that the midwives rounded off the values to the nearest unit. The lack of association between cognitive performance and birth size measurements in some of the studies in this review may be because of this inaccuracy. This was specifically examined in Martyn et al and there was evidence of clumping of the data points suggesting rounding off values.

Five studies^14^, ^15^, ^16^, ^19^, ^21^ did not adjust the analyses for gestational age, which reduces the specificity of birth weight as a measure of fetal growth. This may have resulted in the lack of associations in some of these studies. Most studies did not provide justifications for the majority of adjustments (Table 2). Furthermore, one study^15^ adjusted for a total of 21 different measures (not including gestational age), which makes it difficult to assess how far participants represent the general population. Some studies also did not provide any unadjusted information, making it difficult to assess the role of covariates in the reported effect (Table 2). Depression is related to both size at birth and cognitive function,^34^ but the confounding effect of depression on the association of size at birth with cognitive ability was measured only in one study^20^ in this review.

In this review, most studies report associations of multiple parameters of birth size with multiple cognitive tests, measuring different cognitive domains. While this allows for a comprehensive overview of a variety of cognitive assessments, some significant associations may have resulted from chance alone (risk of type I errors) and or because of multiple testing. For example, in Erickson et al, birth weight was associated with serial sevens test score (a single item from MMSE), though there were no significant associations with 12 cognitive function outcomes, including total MMSE score.

### 5.3 Strengths and limitations of the review process

This review strictly adhered to the study protocol, which was developed prior to the formal search. The forward citation search and reference list search were conducted systematically. Several authors of potentially eligible study were contacted for additional information. All relevant studies appear to have been included in this review. There were no restrictions on publication language, and full‐texts of all potentially relevant articles were evaluated against the inclusion criteria. However, the grey literature was not systematically searched and this may have resulted in non‐identification of potentially relevant studies. Furthermore, it is possible that there are unpublished studies that were not available.

A limitation of this review was that only a small number of eligible studies were retrieved, and it was not possible to conduct meta‐analyses for summary statistics due to heterogeneity. There was considerable heterogeneity across studies, and this is both strength and a weakness. This was expected, given the range of different factors known to contribute to both birth size and late life cognition, the different cognitive tests with their own scoring systems, and the range of demographics across each study. Though most studies from this review indicate that small size at birth is a risk factor for reduced cognitive ability in late life, the clinical relevance of the findings is limited as they do not include outcomes like cognitive impairment and dementia. The generalisability of findings from this review is mostly limited to higher income settings, and there is an urgent need for similar studies in LMIC settings where the burden of both low birth weight and dementia is highest.^26^

### 5.4 Implications: clinical, public health, and future research

In some studies in this review, relatively lower overall effect sizes and a lack of substantial association between birth size and cognitive function in late life may be a reflection of a diminished impact of early factors, as other factors that mitigated these initial differences and reduced or eliminated their influence in later adult life come into play^3^, ^11^; these may include later nutrition, education and occupation status.^3^, ^28^ Both birth weight and socioeconomic position in early life are associated with cognitive function in childhood and adulthood, although postnatal growth and development is thought to be more important than prenatal factors.^7^, ^8^ Cognition in late life is impacted by a cumulative effect of nutrition, education, social, and family environment in early and midlife.^3^ Therefore, there is definitely a need for more research with a lifecourse approach while examining the relationship between birth size and late life cognitive ability. The mediating or confounding effect of childhood growth and development, education, cardiometabolic risk factors, depression, and socioeconomic position should be explored to better understand the lifecourse pathways to cognitive ageing. Further, there is a need for studies examining the underlying mechanisms (for eg, neuroimaging, genetic, and epigenetic studies) linking early life nutrition to cognitive ageing.

Despite these limitations, the findings from this review may support the scientific rationale for improving maternal nutrition, best indicated by the off‐spring birth size, which is known to persistent effect on brain ageing. Recent improvements in the nutrition and education of both mothers and children is likely to produce younger generations with better cognitive health compared with the generation of older adults examined in this review. There is evidence of such an improvement in cognitive function and IQ scores across the generations, mainly from high‐income countries. This is termed the Flynn effect.^35^ A comparison of scores from identical cognitive tests administered to adults 10 to 30 years apart has shown an increase of about five to nine IQ points per decade, and an increase of about five to 25 IQ points over a generation.^35^ Further, a rise in intelligence scores of about 12 IQ points over a period of 50 years or two IQ points per decade has also been observed in the UK cohorts.^36^ The reasons for such improvements in cognition and IQ across the generations are not well understood. Possible mechanisms include improvements in nutrition in early life and childhood, improvements in educational standards and schooling, improvements in technology leading to easier access to information, and perhaps increased complexity of the environment. Thus, the existence of a Flynn effect, though not fully proven, suggests that intelligence is not a fixed genetic attribute but is modifiable by the environment.

## 6 CONCLUSIONS

Most studies in this review indicate that smaller size at birth is a risk factor for lower cognitive function in late life, at least in higher income countries. It was not possible to conduct meta‐analyses for summary statistics due to clinical heterogeneity. While the aim of assessing the association of birth size with cognitive ability in late life is to draw conclusions about the relationship between the prenatal environment and later cognitive outcomes, such definitive conclusions cannot be drawn from birth size data alone. Future research should take a considered approach to covariates across the life course and explore pathways for cognitive ageing.

## CONFLICT OF INTEREST

None of the authors declare any conflict of interest.

## AUTHOR CONTRIBUTIONS

The study was designed by M.K., C.H.D.F., and K.K. The literature search and data extraction was conducted by S.J., B.D.U., M.M., and M.K. The project was supported and supervised by S.C.K.

## APPENDIX A

### Search strategy from MEDLINE.

1. MEDLINE; exp BIRTH WEIGHT/; 34390 results.
2. MEDLINE; (birth adj5 length).ti,ab; 3033 results.
3. MEDLINE; (birth adj5 circumference).ti,ab; 1149 results.
4. MEDLINE; "ponderal index".ti,ab; 938 results.
5. MEDLINE; exp INFANT, SMALL FOR GESTATIONAL AGE/; 5360 results.
6. MEDLINE; "small for gestational age".ti,ab; 6414 results.
7. MEDLINE; "foetal origins hypothesis".ti,ab; 15 results.
8. MEDLINE; "fetal origins hypothesis".ti,ab; 103 results.
9. MEDLINE; "growth in utero".ti,ab; 178 results.
10. MEDLINE; exp FETAL DEVELOPMENT/; 75682 results.
11. MEDLINE; "fetal growth".ti,ab; 10233 results.
12. MEDLINE; "foetal growth".ti,ab; 592 results.
13. MEDLINE; exp FETAL GROWTH RETARDATION/; 13101 results.
14. MEDLINE; "intrauterine growth".ti,ab; 10002 results.
15. MEDLINE; (prenatal adj5 undernutrition).ti,ab; 134 results.
16. MEDLINE; (birth adj5 size).ti,ab; 2958 results.
17. MEDLINE; 1 OR 2 OR 3 OR 4 OR 5 OR 6 OR 7 OR 8 OR 9 OR 10 OR 11 OR 12 OR 13 OR 14 OR 15 OR 16; 121676 results.
18. MEDLINE; exp COGNITION/; 112890 results.
19. MEDLINE; exp MEMORY/; 102877 results.
20. MEDLINE; exp MENTAL RECALL/; 27549 results.
21. MEDLINE; exp ATTENTION/; 61050 results.
22. MEDLINE; cognition.ti,ab; 35514 results.
23. MEDLINE; memory.ti,ab; 163113 results.
24. MEDLINE; recall.ti,ab; 37487 results.
25. MEDLINE; attention.ti,ab; 251466 results.
26. MEDLINE; 18 OR 19 OR 20 OR 21 OR 22 OR 23 OR 24 OR 25; 576383 results.
27. MEDLINE; 17 AND 26; 2300 results.
28. MEDLINE; 27 [Limit to: (Age Groups Middle Aged 45 plus years or All Aged 65 and Over or Aged 80 and Over)]; 99 results

## APPENDIX B

### STROBE checklist for studies included in the systematic review.

**Table:**

	Item No	Recommendation	Page Number
Title and abstract	1	(a) Indicate the study's design with a commonly used term in the title or the abstract.	1
		(b) Provide in the abstract an informative and balanced summary of what was done and what was found.	1
Introduction			
Background/rationale	2	Explain the scientific background and rationale for the investigation being reported.	1
Objectives	3	State specific objectives, including any pre‐specified hypotheses.	1
Methods			
Study design	4	Present key elements of study design early in the paper.	1‐2
Setting	5	Describe the setting, locations, and relevant dates, including periods of recruitment, exposure, follow‐up, and data collection.	2
Participants	6	(a) *Cohort study*. Give the eligibility criteria and the sources and methods of the selection of participants. Describe methods of follow‐up. *Case‐control study*. Give the eligibility criteria and the sources and methods of case ascertainment and control selection. Give the rationale for the choice of cases and controls. *Cross‐sectional study*. Give the eligibility criteria and the sources and methods of selection of participants.	2
		(b) *Cohort study*. For matched studies, give matching criteria and number of exposed and unexposed. *Case‐control study*. For matched studies, give matching criteria and the number of controls per case.	
Variables	7	Clearly define all outcomes, exposures, predictors, potential confounders, and effect modifiers. Give diagnostic criteria, if applicable.	2‐3
Data sources/measurement	8*	For each variable of interest, give sources of data and details of methods of assessment (measurement). Describe comparability of assessment methods if there is more than one group.	2‐3
Bias	9	Describe any efforts to address potential sources of bias.	5
Study size	10	Explain how the study size was arrived at.	no
Quantitative variables	11	Explain how quantitative variables were handled in the analyses. If applicable, describe which groupings were chosen and why.	3‐4
Statistical methods	12	(a) Describe all statistical methods, including those used to control for confounding.	2‐4
		(b) Describe any methods used to examine subgroups and interactions.	2‐4
		(c) Explain how missing data were addressed.	no
		(d) *Cohort study*. If applicable, explain how loss to follow‐up was addressed. *Case‐control study*. If applicable, explain how matching of cases and controls was addressed. *Cross‐sectional study*. If applicable, describe analytical methods taking account of sampling strategy.	
		(e) Describe any sensitivity analyses.	no
Results			
Participants	13*	(a) Report numbers of individuals at each stage of study—eg, numbers potentially eligible, examined for eligibility, confirmed eligible, included in the study, completing follow‐up, and analysed.	3
		(b) Give reasons for non‐participation at each stage.	no
		(c) Consider use of a flow diagram.	no
Descriptive data	14*	(a) Give characteristics of study participants (eg, demographic, clinical, social) and information on exposures and potential confounders.	3
		(b) Indicate number of participants with missing data for each variable of interest.	no
		(c) *Cohort study*. Summarise follow‐up time (eg, average and total amount).	2‐3
Outcome data	15*	*Cohort study*. Report numbers of outcome events or summary measures over time.	2
		*Case‐control study*. Report numbers in each exposure category or summary measures of exposure.	
		*Cross‐sectional study*. Report numbers of outcome events or summary measures.	
Main results	16	(a) Give unadjusted estimates and, if applicable, confounder‐adjusted estimates and their precision (eg, 95% confidence interval). Make clear which confounders were adjusted for and why they were included.	4
		(b) Report category boundaries when continuous variables were categorised	
		(c) If relevant, consider translating estimates of relative risk into absolute risk for a meaningful time period.	
Other analyses	17	Report other analyses done—eg, analyses of subgroups and interactions and sensitivity analyses.	4
Discussion			
Key results	18	Summarise key results with reference to study objectives.	3‐4
Limitations	19	Discuss limitations of the study, taking into account sources of potential bias or imprecision. Discuss both direction and magnitude of any potential bias.	4
Interpretation	20	Give a cautious overall interpretation of results considering objectives, limitations, multiplicity of analyses, results from similar studies, and other relevant evidence.	4
Generalisability	21	Discuss the generalisability (external validity) of the study results.	4
Other information			
Funding	22	Give the source of funding and the role of the funders for the present study and, if applicable, for the original study on which the present article is based	4
Title and abstract	1	(a) Indicate the study's design with a commonly used term in the title or the abstract.	1
		(b) Provide in the abstract an informative and balanced summary of what was done and what was found.	1
Introduction			
Background/rationale	2	Explain the scientific background and rationale for the investigation being reported.	2
Objectives	3	State specific objectives, including any prespecified hypotheses.	2
Methods			
Study design	4	Present key elements of study design early in the paper.	2
Setting	5	Describe the setting, locations, and relevant dates, including periods of recruitment, exposure, follow‐up, and data collection.	2
Participants	6	(a) *Cohort study*. Give the eligibility criteria, and the sources and methods of selection of participants. Describe methods of follow‐up. *Case‐control study*. Give the eligibility criteria and the sources and methods of case ascertainment and control selection. Give the rationale for the choice of cases and controls. *Cross‐sectional study*. Give the eligibility criteria and the sources and methods of selection of participants.	2
		(b) *Cohort study*. For matched studies, give matching criteria and number of exposed and unexposed. *Case‐control study*. For matched studies, give matching criteria and the number of controls per case.	
Variables	7	Clearly define all outcomes, exposures, predictors, potential confounders, and effect modifiers. Give diagnostic criteria, if applicable.	3
Data sources/measurement	8*	For each variable of interest, give sources of data and details of methods of assessment (measurement). Describe comparability of assessment methods if there is more than one group.	3
Bias	9	Describe any efforts to address potential sources of bias.	2‐3
Study size	10	Explain how the study size was arrived at.	no
Quantitative variables	11	Explain how quantitative variables were handled in the analyses. If applicable, describe which groupings were chosen and why.	3
Statistical methods	12	(a) Describe all statistical methods, including those used to control for confounding.	3‐5
		(b) Describe any methods used to examine subgroups and interactions.	5
		(c) Explain how missing data were addressed.	no
		(d) *Cohort study*. If applicable, explain how loss to follow‐up was addressed. *Case‐control study*. If applicable, explain how matching of cases and controls was addressed. *Cross‐sectional study*. If applicable, describe analytical methods taking account of sampling strategy.	no
		(e) Describe any sensitivity analyses.	5
Results			
Participants	13*	(a) Report numbers of individuals at each stage of study—eg, numbers potentially eligible, examined for eligibility, confirmed eligible, included in the study, completing follow‐up, and analysed.	2
		(b) Give reasons for non‐participation at each stage.	2
		(c) Consider use of a flow diagram.	no
Descriptive data	14*	(a) Give characteristics of study participants (eg, demographic, clinical, social) and information on exposures and potential confounders.	3
		(b) Indicate number of participants with missing data for each variable of interest.	3
		(c) *Cohort study*. Summarise follow‐up time (eg, average and total amount).	3
Outcome data	15*	*Cohort study*. Report numbers of outcome events or summary measures over time.	2
		*Case‐control study*. Report numbers in each exposure category or summary measures of exposure.	
		*Cross‐sectional study*. Report numbers of outcome events or summary measures.	
Main results	16	(a) Give unadjusted estimates and, if applicable, confounder‐adjusted estimates and their precision (eg, 95% confidence interval). Make clear which confounders were adjusted for and why they were included.	3‐5
		(b) Report category boundaries when continuous variables were categorised.	
		(c) If relevant, consider translating estimates of relative risk into absolute risk for a meaningful time period.	
Other analyses	17	Report other analyses done—eg, analyses of subgroups and interactions and sensitivity analyses.	3‐5
Discussion			
Key results	18	Summarise key results with reference to study objectives.	6
Limitations	19	Discuss limitations of the study, taking into account sources of potential bias or imprecision. Discuss both direction and magnitude of any potential bias.	6
Interpretation	20	Give a cautious overall interpretation of results considering objectives, limitations, multiplicity of analyses, results from similar studies, and other relevant evidence.	6
Generalisability	21	Discuss the generalisability (external validity) of the study results.	no
Other information			
Funding	22	Give the source of funding and the role of the funders for the present study and, if applicable, for the original study on which the present article is based.	7

	Item No	Recommendation	Page Number
Title and abstract	1	(a) Indicate the study's design with a commonly used term in the title or the abstract.	1
		(b) Provide in the abstract an informative and balanced summary of what was done and what was found.	1
Introduction			
Background/rationale	2	Explain the scientific background and rationale for the investigation being reported.	2
Objectives	3	State specific objectives, including any prespecified hypotheses.	2
Methods			
Study design	4	Present key elements of study design early in the paper.	2‐3
Setting	5	Describe the setting, locations, and relevant dates, including periods of recruitment, exposure, follow‐up, and data collection	2‐3
Participants	6	(*a*) *Cohort study*. Give the eligibility criteria and the sources and methods of selection of participants. Describe methods of follow‐up. *Case‐control study*. Give the eligibility criteria and the sources and methods of case ascertainment and control selection. Give the rationale for the choice of cases and controls. *Cross‐sectional study*. Give the eligibility criteria and the sources and methods of selection of participants.	2‐3
		(b) *Cohort study*. For matched studies, give matching criteria and number of exposed and unexposed. *Case‐control study* For matched studies, give matching criteria and the number of controls per case.	
Variables	7	Clearly define all outcomes, exposures, predictors, potential confounders, and effect modifiers. Give diagnostic criteria, if applicable.	2‐3
Data sources/measurement	8*	For each variable of interest, give sources of data and details of methods of assessment (measurement). Describe comparability of assessment methods if there is more than one group.	2‐3
Bias	9	Describe any efforts to address potential sources of bias.	
Study size	10	Explain how the study size was arrived at.	
Quantitative variables	11	Explain how quantitative variables were handled in the analyses. If applicable, describe which groupings were chosen and why.	4‐6
Statistical methods	12	(a) Describe all statistical methods, including those used to control for confounding.	4‐6
		(b) Describe any methods used to examine subgroups and interactions.	4‐6
		(c) Explain how missing data were addressed.	no
		(d) *Cohort study*. If applicable, explain how loss to follow‐up was addressed. *Case‐control study*. If applicable, explain how matching of cases and controls was addressed. *Cross‐sectional study*. If applicable, describe analytical methods taking account of sampling strategy.	no
		(e) Describe any sensitivity analyses.	4‐6
Results			
Participants	13*	(a) Report numbers of individuals at each stage of study—eg, numbers potentially eligible, examined for eligibility, confirmed eligible, included in the study, completing follow‐up, and analysed.	2
		(b) Give reasons for non‐participation at each stage.	no
		(c) Consider use of a flow diagram.	no
Descriptive data	14*	(a) Give characteristics of study participants (eg, demographic, clinical, and social) and information on exposures and potential confounders.	4
		(b) Indicate number of participants with missing data for each variable of interest.	
		(c) *Cohort study*. Summarise follow‐up time (eg, average and total amount)	2
Outcome data	15*	*Cohort study*. Report numbers of outcome events or summary measures over time.	4‐6
		*Case‐control study*. Report numbers in each exposure category or summary measures of exposure.	
		*Cross‐sectional study*. Report numbers of outcome events or summary measures.	
Main results	16	(a) Give unadjusted estimates and, if applicable, confounder‐adjusted estimates and their precision (eg, 95% confidence interval). Make clear which confounders were adjusted for and why they were included.	5‐7
		(b) Report category boundaries when continuous variables were categorised.	5‐7
		(c) If relevant, consider translating estimates of relative risk into absolute risk for a meaningful time period.	no
Other analyses	17	Report other analyses done—eg, analyses of subgroups and interactions and sensitivity analyses.	6‐7
Discussion			
Key results	18	Summarise key results with reference to study objectives.	6
Limitations	19	Discuss limitations of the study, taking into account sources of potential bias or imprecision. Discuss both direction and magnitude of any potential bias.	6
Interpretation	20	Give a cautious overall interpretation of results considering objectives, limitations, multiplicity of analyses, results from similar studies, and other relevant evidence.	6‐7
Generalisability	21	Discuss the generalisability (external validity) of the study results.	16
Other information			
Funding	22	Give the source of funding and the role of the funders for the present study and, if applicable, for the original study on which the present article is based.	10

	Item No	Recommendation	Page Number
Title and abstract	1	(a) Indicate the study's design with a commonly used term in the title or the abstract.	1
		(b) Provide in the abstract an informative and balanced summary of what was done and what was found.	1
Introduction			
Background/rationale	2	Explain the scientific background and rationale for the investigation being reported.	1‐2
Objectives	3	State specific objectives, including any prespecified hypotheses.	2
Methods			
Study design	4	Present key elements of study design early in the paper.	2
Setting	5	Describe the setting, locations, and relevant dates, including periods of recruitment, exposure, follow‐up, and data collection.	2
Participants	6	(a) *Cohort study*. Give the eligibility criteria and the sources and methods of selection of participants. Describe methods of follow‐up. *Case‐control study*. Give the eligibility criteria and the sources and methods of case ascertainment and control selection. Give the rationale for the choice of cases and controls. *Cross‐sectional study*. Give the eligibility criteria and the sources and methods of selection of participants.	2
		(b) *Cohort study*. For matched studies, give matching criteria and number of exposed and unexposed. *Case‐control study*. For matched studies, give matching criteria and the number of controls per case.	2
Variables	7	Clearly define all outcomes, exposures, predictors, potential confounders, and effect modifiers. Give diagnostic criteria, if applicable.	2‐3
Data sources/measurement	8*	For each variable of interest, give sources of data and details of methods of assessment (measurement). Describe comparability of assessment methods if there is more than one group.	2‐3
Bias	9	Describe any efforts to address potential sources of bias.	3
Study size	10	Explain how the study size was arrived at.	no
Quantitative variables	11	Explain how quantitative variables were handled in the analyses. If applicable, describe which groupings were chosen and why.	3‐5
Statistical methods	12	(a) Describe all statistical methods, including those used to control for confounding.	5‐6
		(b) Describe any methods used to examine subgroups and interactions.	no
		(c) Explain how missing data were addressed.	no
		(d) *Cohort study*. If applicable, explain how loss to follow‐up was addressed. *Case‐control study*. If applicable, explain how matching of cases and controls was addressed. *Cross‐sectional study*. If applicable, describe analytical methods taking account of sampling strategy.	no
		(e) Describe any sensitivity analyses.	yes
Results			
Participants	13*	(a) Report numbers of individuals at each stage of study—eg, numbers potentially eligible, examined for eligibility, confirmed eligible, included in the study, completing follow‐up, and analysed.	2
		(b) Give reasons for non‐participation at each stage.	2
		(c) Consider use of a flow diagram.	no
Descriptive data	14*	(a) Give characteristics of study participants (eg, demographic, clinical, and social) and information on exposures and potential confounders.	3
		(b) Indicate number of participants with missing data for each variable of interest.	no
		(c) *Cohort study*. Summarise follow‐up time (eg, average and total amount)	na
Outcome data	15*	*Cohort study*. Report numbers of outcome events or summary measures over time.	4‐6
		*Case‐control study*. Report numbers in each exposure category or summary measures of exposure.	
		*Cross‐sectional study*. Report numbers of outcome events or summary measures.	
Main results	16	(a) Give unadjusted estimates and, if applicable, confounder‐adjusted estimates and their precision (eg, 95% confidence interval). Make clear which confounders were adjusted for and why they were included.	4‐6
		(b) Report category boundaries when continuous variables were categorised.	4‐6
		(c) If relevant, consider translating estimates of relative risk into absolute risk for a meaningful time period.	no
Other analyses	17	Report other analyses done—eg, analyses of subgroups and interactions and sensitivity analyses.	4‐6
Discussion			**5‐6**
Key results	18	Summarise key results with reference to study objectives.	6‐7
Limitations	19	Discuss limitations of the study, taking into account sources of potential bias or imprecision. Discuss both direction and magnitude of any potential bias.	6‐7
Interpretation	20	Give a cautious overall interpretation of results considering objectives, limitations, multiplicity of analyses, results from similar studies, and other relevant evidence.	6‐7
Generalisability	21	Discuss the generalisability (external validity) of the study results.	8
Other information			
Funding	22	Give the source of funding and the role of the funders for the present study and, if applicable, for the original study on which the present article is based.	8

	Item No	Recommendation	Page Number
Title and abstract	1	(a) Indicate the study's design with a commonly used term in the title or the abstract.	1
		(b) Provide in the abstract an informative and balanced summary of what was done and what was found.	1
Introduction			
Background/rationale	2	Explain the scientific background and rationale for the investigation being reported.	1
Objectives	3	State specific objectives, including any prespecified hypotheses.	1
Methods			
Study design	4	Present key elements of study design early in the paper.	2
Setting	5	Describe the setting, locations, and relevant dates, including periods of recruitment, exposure, follow‐up, and data collection.	2
Participants	6	(a) *Cohort study*. Give the eligibility criteria and the sources and methods of selection of participants. Describe methods of follow‐up. *Case‐control study*. Give the eligibility criteria and the sources and methods of case ascertainment and control selection. Give the rationale for the choice of cases and controls. *Cross‐sectional study*. Give the eligibility criteria and the sources and methods of selection of participants.	2‐3
		(b) *Cohort study*. For matched studies, give matching criteria and number of exposed and unexposed. *Case‐control study*. For matched studies, give matching criteria and the number of controls per case.	4
Variables	7	Clearly define all outcomes, exposures, predictors, potential confounders, and effect modifiers. Give diagnostic criteria, if applicable.	4‐5
Data sources/measurement	8*	For each variable of interest, give sources of data and details of methods of assessment (measurement). Describe comparability of assessment methods if there is more than one group.	4‐5
Bias	9	Describe any efforts to address potential sources of bias.	4
Study size	10	Explain how the study size was arrived at.	no
Quantitative variables	11	Explain how quantitative variables were handled in the analyses. If applicable, describe which groupings were chosen and why.	4‐5
Statistical methods	12	(a) Describe all statistical methods, including those used to control for confounding.	4‐5
		(b) Describe any methods used to examine subgroups and interactions.	no
		(c) Explain how missing data were addressed.	no
		(d) *Cohort study*. If applicable, explain how loss to follow‐up was addressed. *Case‐control study*. If applicable, explain how matching of cases and controls was addressed. *Cross‐sectional study*. If applicable, describe analytical methods taking account of sampling strategy.	
		(e) Describe any sensitivity analyses.	4‐5
Results			
Participants	13*	(a) Report numbers of individuals at each stage of study—eg, numbers potentially eligible, examined for eligibility, confirmed eligible, included in the study, completing follow‐up, and analysed.	2‐4
		(b) Give reasons for non‐participation at each stage.	2‐4
		(c) Consider use of a flow diagram.	no
Descriptive data	14*	(a) Give characteristics of study participants (eg, demographic, clinical, and social) and information on exposures and potential confounders.	2
		(b) Indicate number of participants with missing data for each variable of interest.	no
		(c) *Cohort study*. Summarise follow‐up time (eg, average and total amount).	4
Outcome data	15*	*Cohort study*. Report numbers of outcome events or summary measures over time.	5
		*Case‐control study*. Report numbers in each exposure category, or summary measures of exposure.	
		*Cross‐sectional study*. Report numbers of outcome events or summary measures.	
Main results	16	(a) Give unadjusted estimates and, if applicable, confounder‐adjusted estimates and their precision (eg, 95% confidence interval). Make clear which confounders were adjusted for and why they were included.	4‐5
		(b) Report category boundaries when continuous variables were categorised.	
		(c) If relevant, consider translating estimates of relative risk into absolute risk for a meaningful time period.	4‐5
Other analyses	17	Report other analyses done—eg, analyses of subgroups and interactions and sensitivity analyses	3
Discussion			
Key results	18	Summarise key results with reference to study objectives.	3
Limitations	19	Discuss limitations of the study, taking into account sources of potential bias or imprecision. Discuss both direction and magnitude of any potential bias.	4
Interpretation	20	Give a cautious overall interpretation of results considering objectives, limitations, multiplicity of analyses, results from similar studies, and other relevant evidence.	4
Generalisability	21	Discuss the generalisability (external validity) of the study results.	no
Other information			
Funding	22	Give the source of funding and the role of the funders for the present study and, if applicable, for the original study on which the present article is based.	5

	Item No	Recommendation	Page Number
Title and abstract	1	(a) Indicate the study's design with a commonly used term in the title or the abstract.	1
		(b) Provide in the abstract an informative and balanced summary of what was done and what was found.	1
Introduction			
Background/rationale	2	Explain the scientific background and rationale for the investigation being reported.	1‐2
Objectives	3	State specific objectives, including any prespecified hypotheses.	1‐2
Methods			
Study design	4	Present key elements of study design early in the paper.	1‐2
Setting	5	Describe the setting, locations, and relevant dates, including periods of recruitment, exposure, follow‐up, and data collection.	2
Participants	6	(a) *Cohort study*. Give the eligibility criteria and the sources and methods of selection of participants. Describe methods of follow‐up. *Case‐control study*. Give the eligibility criteria and the sources and methods of case ascertainment and control selection. Give the rationale for the choice of cases and controls. *Cross‐sectional study*. Give the eligibility criteria and the sources and methods of selection of participants	2
		(b) *Cohort study*. For matched studies, give matching criteria and number of exposed and unexposed. *Case‐control study*. For matched studies, give matching criteria and the number of controls per case.	
Variables	7	Clearly define all outcomes, exposures, predictors, potential confounders, and effect modifiers. Give diagnostic criteria, if applicable.	2
Data sources/measurement	8*	For each variable of interest, give sources of data and details of methods of assessment (measurement). Describe comparability of assessment methods if there is more than one group.	2
Bias	9	Describe any efforts to address potential sources of bias.	2
Study size	10	Explain how the study size was arrived at.	no
Quantitative variables	11	Explain how quantitative variables were handled in the analyses. If applicable, describe which groupings were chosen and why.	3
Statistical methods	12	(a) Describe all statistical methods, including those used to control for confounding.	3‐4
		(b) Describe any methods used to examine subgroups and interactions.	no
		(c) Explain how missing data were addressed.	no
		(d) *Cohort study*. If applicable, explain how loss to follow‐up was addressed. *Case‐control study*. If applicable, explain how matching of cases and controls was addressed. *Cross‐sectional study*. If applicable, describe analytical methods taking account of sampling strategy.	
		(e) Describe any sensitivity analyses.	no
Results			
Participants	13*	(a) Report numbers of individuals at each stage of study—eg, numbers potentially eligible, examined for eligibility, confirmed eligible, included in the study, completing follow‐up, and analysed.	2‐4
		(b) Give reasons for non‐participation at each stage.	no
		(c) Consider use of a flow diagram.	no
Descriptive data	14*	(a) Give characteristics of study participants (eg, demographic, clinical, and social) and information on exposures and potential confounders.	4
		(b) Indicate number of participants with missing data for each variable of interest.	
		(c) *Cohort study*. Summarise follow‐up time (eg, average and total amount).	4
Outcome data	15*	*Cohort study*. Report numbers of outcome events or summary measures over time.	
		*Case‐control study*. Report numbers in each exposure category or summary measures of exposure.	*4*
		*Cross‐sectional study*. Report numbers of outcome events or summary measures.	
Main results	16	(a) Give unadjusted estimates and, if applicable, confounder‐adjusted estimates and their precision (eg, 95% confidence interval). Make clear which confounders were adjusted for and why they were included.	5
		(b) Report category boundaries when continuous variables were categorised.	
		(c) If relevant, consider translating estimates of relative risk into absolute risk for a meaningful time period.	
Other analyses	17	Report other analyses done—eg, analyses of subgroups and interactions and sensitivity analyses.	5
Discussion			
Key results	18	Summarise key results with reference to study objectives.	3
Limitations	19	Discuss limitations of the study, taking into account sources of potential bias or imprecision. Discuss both direction and magnitude of any potential bias.	5
Interpretation	20	Give a cautious overall interpretation of results considering objectives, limitations, multiplicity of analyses, results from similar studies, and other relevant evidence.	5
Generalisability	21	Discuss the generalisability (external validity) of the study results.	no
Other information			
Funding	22	Give the source of funding and the role of the funders for the present study and, if applicable, for the original study on which the present article is based.	6

	Item No	Recommendation	Page Number
Title and abstract	1	(a) Indicate the study's design with a commonly used term in the title or the abstract.	1
		(b) Provide in the abstract an informative and balanced summary of what was done and what was found.	1
Introduction			
Background/rationale	2	Explain the scientific background and rationale for the investigation being reported.	1
Objectives	3	State specific objectives, including any prespecified hypotheses.	2
Methods			
Study design	4	Present key elements of study design early in the paper.	2
Setting	5	Describe the setting, locations, and relevant dates, including periods of recruitment, exposure, follow‐up, and data collection.	2
Participants	6	(a) *Cohort study*. Give the eligibility criteria and the sources and methods of selection of participants. Describe methods of follow‐up. *Case‐control study*. Give the eligibility criteria and the sources and methods of case ascertainment and control selection. Give the rationale for the choice of cases and controls. *Cross‐sectional study*. Give the eligibility criteria and the sources and methods of selection of participants.	2
		(b) *Cohort study*. For matched studies, give matching criteria and number of exposed and unexposed. *Case‐control study*. For matched studies, give matching criteria and the number of controls per case.	
Variables	7	Clearly define all outcomes, exposures, predictors, potential confounders, and effect modifiers. Give diagnostic criteria, if applicable.	2
Data sources/measurement	8*	For each variable of interest, give sources of data and details of methods of assessment (measurement). Describe comparability of assessment methods if there is more than one group.	2
Bias	9	Describe any efforts to address potential sources of bias.	2
Study size	10	Explain how the study size was arrived at.	no
Quantitative variables	11	Explain how quantitative variables were handled in the analyses. If applicable, describe which groupings were chosen and why.	2‐3
Statistical methods	12	(a) Describe all statistical methods, including those used to control for confounding.	2‐3
		(b) Describe any methods used to examine subgroups and interactions.	3
		(c) Explain how missing data were addressed.	3
		(d) *Cohort study*. If applicable, explain how loss to follow‐up was addressed. *Case‐control study*. If applicable, explain how matching of cases and controls was addressed. *Cross‐sectional study*. If applicable, describe analytical methods taking account of sampling strategy.	
		(e) Describe any sensitivity analyses.	no
Results			3
Participants	13*	(a) Report numbers of individuals at each stage of study—eg, numbers potentially eligible, examined for eligibility, confirmed eligible, included in the study, completing follow‐up, and analysed.	2‐3
		(b) Give reasons for non‐participation at each stage.	no
		(c) Consider use of a flow diagram.	3
Descriptive data	14*	(a) Give characteristics of study participants (eg, demographic, clinical, and social) and information on exposures and potential confounders.	3
		(b) Indicate number of participants with missing data for each variable of interest.	
		(c) *Cohort study*. Summarise follow‐up time (eg, average and total amount).	
Outcome data	15*	*Cohort study*. Report numbers of outcome events or summary measures over time.	3
		*Case‐control study*. Report numbers in each exposure category, or summary measures of exposure.	
		*Cross‐sectional study*. Report numbers of outcome events or summary measures.	
Main results	16	(a) Give unadjusted estimates and, if applicable, confounder‐adjusted estimates and their precision (eg, 95% confidence interval). Make clear which confounders were adjusted for and why they were included.	4
		(b) Report category boundaries when continuous variables were categorised.	
		(c) If relevant, consider translating estimates of relative risk into absolute risk for a meaningful time period.	
Other analyses	17	Report other analyses done—eg, analyses of subgroups and interactions and sensitivity analyses.	4
Discussion			
Key results	18	Summarise key results with reference to study objectives.	4
Limitations	19	Discuss limitations of the study, taking into account sources of potential bias or imprecision. Discuss both direction and magnitude of any potential bias.	5
Interpretation	20	Give a cautious overall interpretation of results considering objectives, limitations, multiplicity of analyses, results from similar studies, and other relevant evidence.	5
Generalisability	21	Discuss the generalisability (external validity) of the study results.	no
Other information			
Funding	22	Give the source of funding and the role of the funders for the present study and, if applicable, for the original study on which the present article is based.	5

	Item No	Recommendation	Page Number
Title and abstract	1	(a) Indicate the study's design with a commonly used term in the title or the abstract.	1
		(b) Provide in the abstract an informative and balanced summary of what was done and what was found.	1
Introduction			
Background/rationale	2	Explain the scientific background and rationale for the investigation being reported.	1‐2
Objectives	3	State specific objectives, including any prespecified hypotheses.	2
Methods			
Study design	4	Present key elements of study design early in the paper.	2
Setting	5	Describe the setting, locations, and relevant dates, including periods of recruitment, exposure, follow‐up, and data collection.	2
Participants	6	(a) *Cohort study*. Give the eligibility criteria and the sources and methods of selection of participants. Describe methods of follow‐up. *Case‐control study*. Give the eligibility criteria and the sources and methods of case ascertainment and control selection. Give the rationale for the choice of cases and controls. *Cross‐sectional study*. Give the eligibility criteria and the sources and methods of selection of participants.	2
		(b) *Cohort study*. For matched studies, give matching criteria and number of exposed and unexposed. *Case‐control study*. For matched studies, give matching criteria and the number of controls per case.	
Variables	7	Clearly define all outcomes, exposures, predictors, potential confounders, and effect modifiers. Give diagnostic criteria, if applicable.	2
Data sources/measurement	8*	For each variable of interest, give sources of data and details of methods of assessment (measurement). Describe comparability of assessment methods if there is more than one group.	2
Bias	9	Describe any efforts to address potential sources of bias.	2
Study size	10	Explain how the study size was arrived at.	no
Quantitative variables	11	Explain how quantitative variables were handled in the analyses. If applicable, describe which groupings were chosen and why.	2
Statistical methods	12	(a) Describe all statistical methods, including those used to control for confounding.	2
		(b) Describe any methods used to examine subgroups and interactions.	no
		(c) Explain how missing data were addressed.	2‐3
		(d) *Cohort study*. If applicable, explain how loss to follow‐up was addressed. *Case‐control study*. If applicable, explain how matching of cases and controls was addressed. *Cross‐sectional study*. If applicable, describe analytical methods taking account of sampling strategy.	
		(e) Describe any sensitivity analyses.	no
Results			
Participants	13*	(a) Report numbers of individuals at each stage of study—eg, numbers potentially eligible, examined for eligibility, confirmed eligible, included in the study, completing follow‐up, and analysed.	2
		(b) Give reasons for non‐participation at each stage.	2
		(c) Consider use of a flow diagram.	
Descriptive data	14*	(a) Give characteristics of study participants (eg, demographic, clinical, and social) and information on exposures and potential confounders.	2
		(b) Indicate number of participants with missing data for each variable of interest.	2
		(c) *Cohort study*. Summarise follow‐up time (eg, average and total amount).	
Outcome data	15*	*Cohort study*. Report numbers of outcome events or summary measures over time.	2‐3
		*Case‐control study*. Report numbers in each exposure category or summary measures of exposure.	
		*Cross‐sectional study*. Report numbers of outcome events or summary measures.	
Main results	16	(a) Give unadjusted estimates and, if applicable, confounder‐adjusted estimates and their precision (eg, 95% confidence interval). Make clear which confounders were adjusted for and why they were included.	3‐4
		(b) Report category boundaries when continuous variables were categorised.	
		(c) If relevant, consider translating estimates of relative risk into absolute risk for a meaningful time period.	
Other analyses	17	Report other analyses done—eg, analyses of subgroups and interactions and sensitivity analyses.	3‐4
Discussion			
Key results	18	Summarise key results with reference to study objectives.	4
Limitations	19	Discuss limitations of the study, taking into account sources of potential bias or imprecision. Discuss both direction and magnitude of any potential bias.	5
Interpretation	20	Give a cautious overall interpretation of results considering objectives, limitations, multiplicity of analyses, results from similar studies, and other relevant evidence.	5
Generalisability	21	Discuss the generalisability (external validity) of the study results.	6
Other information			
Funding	22	Give the source of funding and the role of the funders for the present study and, if applicable, for the original study on which the present article is based.	6

	Item No	Recommendation	Page Number
Title and abstract	1	(a) Indicate the study's design with a commonly used term in the title or the abstract.	1
		(b) Provide in the abstract an informative and balanced summary of what was done and what was found.	1
Introduction			
Background/rationale	2	Explain the scientific background and rationale for the investigation being reported.	1
Objectives	3	State specific objectives, including any prespecified hypotheses.	1
Methods			
Study design	4	Present key elements of study design early in the paper.	1
Setting	5	Describe the setting, locations, and relevant dates, including periods of recruitment, exposure, follow‐up, and data collection.	1‐2
Participants	6	(a) *Cohort study*. Give the eligibility criteria and the sources and methods of selection of participants. Describe methods of follow‐up. *Case‐control study*. Give the eligibility criteria and the sources and methods of case ascertainment and control selection. Give the rationale for the choice of cases and controls. *Cross‐sectional study*. Give the eligibility criteria and the sources and methods of selection of participants.	2
		(b) *Cohort study*. For matched studies, give matching criteria and number of exposed and unexposed. *Case‐control study*. For matched studies, give matching criteria and the number of controls per case.	
Variables	7	Clearly define all outcomes, exposures, predictors, potential confounders, and effect modifiers. Give diagnostic criteria, if applicable.	2
Data sources/measurement	8*	For each variable of interest, give sources of data and details of methods of assessment (measurement). Describe comparability of assessment methods if there is more than one group.	2
Bias	9	Describe any efforts to address potential sources of bias.	2
Study size	10	Explain how the study size was arrived at.	no
Quantitative variables	11	Explain how quantitative variables were handled in the analyses. If applicable, describe which groupings were chosen and why.	2
Statistical methods	12	(a) Describe all statistical methods, including those used to control for confounding.	2
		(b) Describe any methods used to examine subgroups and interactions.	no
		(c) Explain how missing data were addressed.	2
		(d) *Cohort study*. If applicable, explain how loss to follow‐up was addressed. *Case‐control study*. If applicable, explain how matching of cases and controls was addressed. *Cross‐sectional study*. If applicable, describe analytical methods taking account of sampling strategy.	
		(e) Describe any sensitivity analyses.	no
Results			
Participants	13*	(a) Report numbers of individuals at each stage of study—eg, numbers potentially eligible, examined for eligibility, confirmed eligible, included in the study, completing follow‐up, and analysed.	1‐2
		(b) Give reasons for non‐participation at each stage.	1‐2
		(c) Consider use of a flow diagram.	no
Descriptive data	14*	(a) Give characteristics of study participants (eg, demographic, clinical, and social) and information on exposures and potential confounders.	2
		(b) Indicate number of participants with missing data for each variable of interest.	2
		(c) *Cohort study*. Summarise follow‐up time (eg, average and total amount).	
Outcome data	15*	*Cohort study*. Report numbers of outcome events or summary measures over time.	2‐3
		*Case‐control study*. Report numbers in each exposure category or summary measures of exposure.	
		*Cross‐sectional study*. Report numbers of outcome events or summary measures.	
Main results	16	(a) Give unadjusted estimates and, if applicable, confounder‐adjusted estimates and their precision (eg, 95% confidence interval). Make clear which confounders were adjusted for and why they were included.	2‐3
		(b) Report category boundaries when continuous variables were categorised.	
		(c) If relevant, consider translating estimates of relative risk into absolute risk for a meaningful time period.	
Other analyses	17	Report other analyses done—eg, analyses of subgroups and interactions and sensitivity analyses.	2‐3
Discussion			
Key results	18	Summarise key results with reference to study objectives.	2
Limitations	19	Discuss limitations of the study, taking into account sources of potential bias or imprecision. Discuss both direction and magnitude of any potential bias.	2
Interpretation	20	Give a cautious overall interpretation of results considering objectives, limitations, multiplicity of analyses, results from similar studies, and other relevant evidence.	3
Generalisability	21	Discuss the generalisability (external validity) of the study results.	no
Other information			
Funding	22	Give the source of funding and the role of the funders for the present study and, if applicable, for the original study on which the present article is based.	4

	Item No	Recommendation	Page Number
Title and abstract	1	(a) Indicate the study's design with a commonly used term in the title or the abstract.	1
		(b) Provide in the abstract an informative and balanced summary of what was done and what was found.	1
Introduction			
Background/rationale	2	Explain the scientific background and rationale for the investigation being reported.	1
Objectives	3	State specific objectives, including any prespecified hypotheses.	2
Methods			
Study design	4	Present key elements of study design early in the paper.	2
Setting	5	Describe the setting, locations, and relevant dates, including periods of recruitment, exposure, follow‐up, and data collection.	2
Participants	6	(a) *Cohort study*. Give the eligibility criteria and the sources and methods of selection of participants. Describe methods of follow‐up. *Case‐control study*. Give the eligibility criteria and the sources and methods of case ascertainment and control selection. Give the rationale for the choice of cases and controls. *Cross‐sectional study*. Give the eligibility criteria and the sources and methods of selection of participants.	
		(b) *Cohort study*. For matched studies, give matching criteria and number of exposed and unexposed. *Case‐control study*. For matched studies, give matching criteria and the number of controls per case.	
Variables	7	Clearly define all outcomes, exposures, predictors, potential confounders, and effect modifiers. Give diagnostic criteria, if applicable.	3
Data sources/measurement	8*	For each variable of interest, give sources of data and details of methods of assessment (measurement). Describe comparability of assessment methods if there is more than one group.	3
Bias	9	Describe any efforts to address potential sources of bias.	3
Study size	10	Explain how the study size was arrived at.	no
Quantitative variables	11	Explain how quantitative variables were handled in the analyses. If applicable, describe which groupings were chosen and why.	3‐4
Statistical methods	12	(a) Describe all statistical methods, including those used to control for confounding.	3‐4
		(b) Describe any methods used to examine subgroups and interactions.	3‐4
		(c) Explain how missing data were addressed.	3‐4
		(d) *Cohort study*. If applicable, explain how loss to follow‐up was addressed. *Case‐control study*. If applicable, explain how matching of cases and controls was addressed. *Cross‐sectional study*. If applicable, describe analytical methods taking account of sampling strategy.	
		(e) Describe any sensitivity analyses.	no
Results			
Participants	13*	(a) Report numbers of individuals at each stage of study—eg, numbers potentially eligible, examined for eligibility, confirmed eligible, included in the study, completing follow‐up, and analysed.	2‐3
		(b) Give reasons for non‐participation at each stage.	2‐3
		(c) Consider use of a flow diagram.	no
Descriptive data	14*	(a) Give characteristics of study participants (eg, demographic, clinical, and social) and information on exposures and potential confounders.	2‐3
		(b) Indicate number of participants with missing data for each variable of interest.	2‐3
		(c) *Cohort study*. Summarise follow‐up time (eg, average and total amount).	
Outcome data	15*	*Cohort study*. Report numbers of outcome events or summary measures over time.	3‐4
		*Case‐control study*. Report numbers in each exposure category or summary measures of exposure.	
		*Cross‐sectional study*. Report numbers of outcome events or summary measures.	*4‐5*
Main results	16	(a) Give unadjusted estimates and, if applicable, confounder‐adjusted estimates and their precision (eg, 95% confidence interval). Make clear which confounders were adjusted for and why they were included.	
		(b) Report category boundaries when continuous variables were categorised.	
		(c) If relevant, consider translating estimates of relative risk into absolute risk for a meaningful time period.	
Other analyses	17	Report other analyses done—eg, analyses of subgroups and interactions and sensitivity analyses.	4‐5
Discussion			
Key results	18	Summarise key results with reference to study objectives.	6
Limitations	19	Discuss limitations of the study, taking into account sources of potential bias or imprecision. Discuss both direction and magnitude of any potential bias.	6
Interpretation	20	Give a cautious overall interpretation of results considering objectives, limitations, multiplicity of analyses, results from similar studies, and other relevant evidence.	6‐7
Generalisability	21	Discuss the generalisability (external validity) of the study results.	no
Other information			
Funding	22	Give the source of funding and the role of the funders for the present study and, if applicable, for the original study on which the present article is based.	7

	Item No	Recommendation	Page Number
Title and abstract	1	(a) Indicate the study's design with a commonly used term in the title or the abstract.	1
		(b) Provide in the abstract an informative and balanced summary of what was done and what was found.	1
Introduction			
Background/rationale	2	Explain the scientific background and rationale for the investigation being reported.	1, 2
Objectives	3	State specific objectives, including any prespecified hypotheses.	2, 3
Methods			
Study design	4	Present key elements of study design early in the paper.	2, 3
Setting	5	Describe the setting, locations, and relevant dates, including periods of recruitment, exposure, follow‐up, and data collection.	2, 3
Participants	6	(a) *Cohort study*. Give the eligibility criteria and the sources and methods of selection of participants. Describe methods of follow‐up. *Case‐control study*. Give the eligibility criteria and the sources and methods of case ascertainment and control selection. Give the rationale for the choice of cases and controls. *Cross‐sectional study*. Give the eligibility criteria and the sources and methods of selection of participants.	2, 3, 4
		(b) *Cohort study*. For matched studies, give matching criteria and number of exposed and unexposed. *Case‐control study*. For matched studies, give matching criteria and the number of controls per case.	
Variables	7	Clearly define all outcomes, exposures, predictors, potential confounders, and effect modifiers. Give diagnostic criteria, if applicable.	3, 4
Data sources/measurement	8*	For each variable of interest, give sources of data and details of methods of assessment (measurement). Describe comparability of assessment methods if there is more than one group.	3, 4
Bias	9	Describe any efforts to address potential sources of bias.	3, 4
Study size	10	Explain how the study size was arrived at.	no
Quantitative variables	11	Explain how quantitative variables were handled in the analyses. If applicable, describe which groupings were chosen and why.	6, 7
Statistical methods	12	(a) Describe all statistical methods, including those used to control for confounding.	6, 7
		(b) Describe any methods used to examine subgroups and interactions.	6, 7
		(c) Explain how missing data were addressed.	
		(d) *Cohort study*. If applicable, explain how loss to follow‐up was addressed. *Case‐control study*. If applicable, explain how matching of cases and controls was addressed. *Cross‐sectional study*. If applicable, describe analytical methods taking account of sampling strategy.	3
		(e) Describe any sensitivity analyses.	no
Results			
Participants	13*	(a) Report numbers of individuals at each stage of study—eg, numbers potentially eligible, examined for eligibility, confirmed eligible, included in the study, completing follow‐up, and analysed.	3, 4
		(b) Give reasons for non‐participation at each stage.	3, 4
		(c) Consider use of a flow diagram.	3
Descriptive data	14*	(a) Give characteristics of study participants (eg, demographic, clinical, and social) and information on exposures and potential confounders.	6
		(b) Indicate number of participants with missing data for each variable of interest.	no
		(c) *Cohort study*. Summarise follow‐up time (eg, average and total amount).	3
Outcome data	15*	*Cohort study*. Report numbers of outcome events or summary measures over time.	3
		*Case‐control study—*Report numbers in each exposure category or summary measures of exposure.	
		*Cross‐sectional study*. Report numbers of outcome events or summary measures.	
Main results	16	(a) Give unadjusted estimates and, if applicable, confounder‐adjusted estimates and their precision (eg, 95% confidence interval). Make clear which confounders were adjusted for and why they were included.	7‐9
		(b) Report category boundaries when continuous variables were categorised.	
		(c) If relevant, consider translating estimates of relative risk into absolute risk for a meaningful time period.	
Other analyses	17	Report other analyses done—eg, analyses of subgroups and interactions and sensitivity analyses.	7‐9
Discussion			
Key results	18	Summarise key results with reference to study objectives.	5‐11
Limitations	19	Discuss limitations of the study, taking into account sources of potential bias or imprecision. Discuss both direction and magnitude of any potential bias.	10
Interpretation	20	Give a cautious overall interpretation of results considering objectives, limitations, multiplicity of analyses, results from similar studies, and other relevant evidence.	10‐11
Generalisability	21	Discuss the generalisability (external validity) of the study results.	no
Other information			
Funding	22	Give the source of funding and the role of the funders for the present study and, if applicable, for the original study on which the present article is based.	11
